# Stimulation and Isolation of *Paraphysoderma sedebokerense* (Blastocladiomycota) Propagules and Their Infection Capacity Toward Their Host Under Different Physiological and Environmental Conditions

**DOI:** 10.3389/fcimb.2019.00072

**Published:** 2019-03-27

**Authors:** Armine Asatryan, Sammy Boussiba, Aliza Zarka

**Affiliations:** Microalgal Biotechnology Laboratory, French Associates Institute for Agriculture and Biotechnology of Drylands, The Jacob Blaustein Institutes for Desert Research, Ben-Gurion University of the Negev, Beer-Sheva, Israel

**Keywords:** propagules, encystment, host-parasite interaction, *Haematocuccus pluvialis*, *Paraphysoderma sedebokerense*, blastocladiomycota, chytrid

## Abstract

*Paraphysoderma sedebokerense (P. sedebokerense)* (Blastocladiomycota) is a facultative pathogenic chytrid that causes irreversible damage to some green microalgae. Specific attacks leading to culture collapse under different conditions have only been described in the lucrative microalga *Haematococcus pluvialis (H. pluvialis)*, while generating biomass for ketocarotenoid astaxanthin production, both indoors and outdoors. In order to manage the infection, parasite propagules (zoospores/amoeboid swarmers), the initiators of the disease, must be studied. Until now, no report on isolated *P. sedebokerense* propagules has been published. Here, we report on a reproducible method for the stimulation of *P. sedebokerense* propagule release and their isolation from fungal cultures in synthetic media and infected *H. pluvialis* cultures, and we further studied their development under different conditions. The isolated propagules featured different spore morphotypes, with coatless spherical spores and amoeboid swarmers being the most dominant in the first pulse of propagule release in both cultures. Inoculating the pure propagules with the host, in both the presence and absence of nitrogen, resulted in epidemic development in both green and red cells; however, in red cells, the epidemic developed more quickly in the presence of nitrogen. Biologically non-active autoclaved host cells were used to distinguish the initial stages of recognition from more progressive stages of the epidemics; on these cells, propagules encysted but did not develop further. These results prove the existence of heat-stable recognition sites on the host and an obligatory signal transduction from the host to support fungal cyst development. The propagule isolation method described herein is a breakthrough that will enable researchers to study the influence of different substances on the propagules, specifically as the initiators of the infection, and thus assist in the management of chytrid diseases. Moreover, it will be useful in studying host-parasite recognition and, therefore, will increase our understanding of the multiple chytrid infections found in nature.

## Introduction

The parasite *Paraphysoderma sedebokerense (P. sedebokerense)* from Blastocladiomycota, isolated and initially characterized in our laboratory by Hoffman et al. (2008), has gained much attention because of its host-specific infection of *Haematococcus pluvialis (H. pluvialis)*, which results in the collapse of the algal cultures and the ultimate decline in the production of industrially important red ketocarotenoid astaxanthin. Recent studies have revealed a new host for *P. sedebokerense*—the green microalga *Scenedesmus dimorphus*—which is an important candidate for biofuel production and is easily subjected to parasitic infection (Letcher et al., 2016). The macroscopic symptoms of this parasite in *H. pluvialis* include flocculation at the initial stages of infection, followed by a change of algal cells' color from green to brown. The microscopic symptoms show propagule attachment to the algal cells at the beginning of infection, and encystment and semitransparent sporangia development at later stages. The algal cells harboring these sporangia gradually lose their cellular contents, ending with a microscopically visible, center-localized brown mass residue inside the empty cells (Hoffman et al., 2008).

Most parasites of freshwater algae are zoosporic true fungi and fungi-like organisms, belonging to the taxonomical groups of Chytridiomycota, Blastocladiomycota, Cryptomycota, Oomycota, Alveolata, and Labyrinthulomycota (Carney and Lane, 2014). Based on their motility, zoospores of true fungi (Chytridiomycota, Blastocladiomycota) are the primary dispersive and infective propagules in aquatic habitats, originating from sporangium production after cyst vegetative growth. Blastocladiomycota zoospores are capable of locomotion, either by a single posteriorly located whiplash flagellum in a water environment, or a wall-less propagule capable of an amoeboid motion on the surface of a substrate, such as a host or a nutrient agar (Strittmatter et al., 2016), as well as on a microscope slide.

Environmental cues governing the mass release of zoospores were so far shown to be species-specific (Pistininzi et al., 2014), and have not been reported for the devastating parasite *P. sedebokerense* or its closely related non-mycelial fungal species. The vegetative growth and sporangium production, which are mandatory stages before zoospore production in all zoosporic fungal species, are highly influenced by nutrient availability, pH, water potential, aeration, moisture, light, temperature, availability of the cations, etc. (Soll and Sonneborn, 1971; Ribeiro, 1978; Cerenius and Söderhäll, 1985; Estrada-Garcia et al., 1989; Serrano et al., 2013; Buller, 2014; Pistininzi et al., 2014). Optimized abiotic factors, either separately or in combination, result in a high frequency of sporangium germination (Pistininzi et al., 2014). For example, Hebert and Kelman (1958), found a cardinal temperature (25–30°C) and pH (6–9) for *Physoderma maydis* (Blastocladiales) sporangium germination (by zoospore), in the presence of light (blue) as a key factor. They also stated that spores could be released to the atmosphere in the presence of moisture and in the absence of CO_2_.

In contrast to the above observations, the *Phytophthora megasperma* (Pythiales) zoospore yield on an agar plate was 10-fold higher under dark conditions than under continuous light illumination (Eye et al., 1978). In addition, younger mycelia gave a higher number of zoospores.

Nutrient shortage in the environment stimulates the emergence of zoospores from sporangia and creates favorable conditions for their survival (Pistininzi et al., 2014), while cooling and flooding boost zoospore release by breaking the rigidity of mature sporangia, due to high water pressure (Ribeiro, 1983; Judelson and Blanco, 2005).

Aforecited study in *P. sedebokerense*, sporangia, under favorable conditions, release amoeboid propagules, capable of moving via pseudopodia, which in a couple of hours recognize, settle, attach, and later encyst on algal host cells. The encysted cell develops a germ tube, which penetrates the host cell wall and develops a highly branched rhizoid system inside the host cytoplasm; the cyst then converts into a thin-walled vegetative sporangium (Hoffman et al., 2008; Letcher et al., 2016; Strittmatter et al., 2016). After 2–3 days in a favorable environment, the mature sporangia, which underwent mitotic divisions, open and release amoeboid swarmers, ~1.7–2.3 μm in size (Letcher et al., 2016). As the epidemic progresses and conditions become unfavorable or stressful, the amoeboid swarmers that were produced at later stages encyst and develop into resting sporangia with a thick wall, a dark opaque appearance, and a swollen bulbed germ tube inserted into the host cell. TEM studies conducted by Letcher et al. (2016) revealed the site of meiosis in the resting sporangium and its ability to release either amoeboid swarmers or flagellated spores. However, cytoplasmic cleavage for flagellated zoospore development was detected by Letcher et al. (2016), but with no success in capturing the motile posteriorly flagellated zoospores that were captured by Strittmatter et al. (2016).

Nonetheless, Strittmatter et al. (2016) demonstrated not only fast moving and fast disappearing (max. 20 min long-lasting cells) posteriorly flagellated zoospores (3 μm) in a 1–2-week-old infected culture, but also a second type of amoeboid cells (~5 μm), in an old infected *H. pluvialis* culture, which they suggested to be most probably the result of the fusion of two amoebae. The transition of fast swimming, posteriorly uniflagellated zoospores into infectious amoeboid swarmers occurs with the formation of intermediate pseudopodial cells (Strittmatter et al., 2016). The existence of a third type of small amoebae, 1.5 μm in size, has also been detected and claimed to be infectious to the host (Strittmatter et al., 2016).

Although a great deal of information from microscopic observations has been accumulated about *P. sedebokerense*, the molecular aspects of its interaction with its host still remain to be determined. To achieve this goal, with advanced molecular tools, obtaining a pure preparation of the infection process initiators is of great importance. Here, we report, for the first time, a method for the isolation of parasite propagules, and we study their development on both their live and biologically inactive hosts, in the presence and absence of nitrogen. The results presented here further extend our understanding of a recognition event between partners and can impact the management not only of fungal infections in the industrially important alga *H. pluvialis*, but also of other zoosporic fungal infections in invertebrates and plants.

## Materials and Methods

### *Paraphysoderma sedebokerense* and *Haematococcus pluvialis* Strains

A *Paraphysoderma sedebokerense* blastoclad isolated culture, registered at MycoBank [MB#561751] and GenBank: EF565163 as “Blastocladiales sp. TJ-2007a” (James et al., 2011; Porter et al., 2011), and a *H. pluvialis* (Chlorophyceae, order Volvocales), 1844 em. Wille 1903, SCCAP K-0084, monoculture from the Scandinavian Culture Center for Algae and Protozoa (SCCAP) at the University of Copenhagen, Denmark were used in this study.

### *P. sedebokerense* Growth Conditions and Media

An axenic blastoclad monoculture was grown in an enriched chytrid growth medium (Gutman et al., 2009), with glucose replaced by sucrose, henceforth called the blastoclad growth medium (BGM). This modified medium was routinely used. The liquid culture was incubated at 30°C on a shaker (145 rpm) under continuous dim white light (15 μmol photons m^−2^ s^−1^) illumination with air enriched with CO_2_ (2 %) at a flow rate of 300 mL min^−1^.

### *H. pluvialis* Growth Conditions and Media

The *H. pluvialis* culture was grown in a modified BG-11 (mBG_11_) medium, as was described in Boussiba and Vonshak (1991), containing 1.5 g L^−1^ NaNO_3_ as the sole nitrogen source; hereafter it will be called the nitrogen-replete (NR) medium. An inoculum of a 7-d-old (stationary phase) culture was used to prepare 100 ml of a culture in 250-mL Erlenmeyer flasks, with an initial cell concentration of 2 × 10^5^ cell mL^−1^ (counted with a hemocytometer). The culture was grown for 7 d, in a shaker (150 rpm) enriched with CO_2_ (200 mL min^−1^), at 25°C and 75–110 μmol photon m^−2^ s^−1^. This “green culture” was routinely used. The “red culture” was obtained in the following way: the green culture was washed twice with sterile DDW, and pelleted cells were resuspended in a nitrogen-deprived (ND) mBG_11_ medium to a final concentration of 2 × 10^5^ cell mL^−1^. The ND medium was prepared by replacing NaNO_3_ in the medium with an equivalent molar concentration of NaCl. This culture was illuminated for 3 d with white LED light with an intensity of 150–170 μmol photon m^−2^ s^−1^, at 25°C. Erlenmeyer flasks (250 ml), with 100 ml of culture were used for this purpose.

### Inactivation of *H. pluvialis* Culture

For this, 100 mL of the green or red culture (in a 250-mL Erlenmeyer flask), obtained as described above, was autoclaved at 121°C, for 20 min.

### Stimulation of Propagule Release From Parasite Culture Alone

The blastoclad culture was grown at a large scale (3–4 L) in the BGM medium, as described above. Once most of the cells reached the sporangium stage (10-d-old culture), the BGM medium was replaced with a propagule stimulation medium (PSM), which is composed of mBG_11_ without sodium nitrate and no added sodium chloride. This culture was subjected to cold-shock, by placing it on ice for about 25–30 min, and then moved to a 25°C incubator shaker, with white LED light illumination of an intensity of 150–170 μmol photon m^−2^ s^−1^, for 4 h. After completing the illumination stage, the LED lamp plate was removed from the shaker, and the shaker was reset to normal blastoclad growth conditions. On the following day, a pulse of propagule release was obtained. After each harvest, fresh PSM was added to the sporangium biomass to enable propagule stimulation for the next harvest. In all, 3–6 harvests, in 12-h intervals, were conducted.

### Stimulation of Propagule Release From Infected Algal Culture

The algal medium of the 3–4-d-old infected *H. pluvialis* culture was replaced with the PSM, as described above for the synthetic culture, while keeping all the other parameters similar to algal infection conditions. In a similar way, the propagule-enriched supernatant was collected 3–5 times, in 12-h intervals for propagule harvesting.

### Propagule Harvesting

Immediately after the propagule release, supernatants deriving from both synthetic and infected *H. pluvialis* cultures were collected and passed through 5.0-μm pore size Polyester Track Etch (PETE) membrane filters (SterliTech Corporation). The filtrate containing propagules was centrifuged at 10,000 g for 5 min, and the pellet was resuspended in a fresh NR mBG_11_ medium for the infection tests and microscopic observations.

### Blastoclad Inoculation of Algal Cultures

Harvested propagules were used to inoculate green or red algal cultures in the NR medium (unless otherwise specified) with the following ratio: 1 mg (wet weight) of *H. pluvialis* green culture equal to 1.1–1.5 × 10^5^ cells mL^−1^ and 0.25 mg (wet weight) collected *P. sedebokerense* propagules (6.74 × 10^6^ cells mL^−1^). The inoculated co-cultures were incubated at 30°C as described above for the pure *P. sedebokerense* cultures. The progress of infection was routinely determined microscopically, mostly by Nile red staining. The cultures were monitored until the control (positive) culture collapsed.

### Nile Red Staining

The infection rate of the cultures was visually determined by staining parasite oil bodies with Nile red (9-diethylamino-5H-benzo [alpha] phenoxazine-5-one), as described previously (Gutman et al., 2009) without a washing step. Infected red or green cultures were stained with 100–200 μg L^−1^ Nile red in 2% DMSO (final concentration) and directly observed under light and fluorescence microscopes, using an excitation wavelength of 450–490 nm and a 520-nm cut-off emission filter.

### Light Microscopy

Samples, mounted on microscope slides, were examined in bright-field (BF) or phase contrast (PC), using either a Zeiss Axioscope or a Zeiss Axio Imager 2 microscope. Pictures were taken with Olympus DP70 or AxioCam Zeiss MRc digital cameras at magnifications of x100, x400, and x1000.

### High-Resolution Scanning Electron Microscopy

The algal, fungal, and infected cells were harvested by centrifugation (MiniSpin Plus Microcentrifuge) at 16,000 g, for 5 min, washed with a phosphate-buffered saline (PBS) solution and fixed with 2.5 % glutaraldehyde (Sigma-Aldrich 340855), at 4°C overnight. The fixed sample was gently rinsed twice with PBS, followed by post-fixation in 1% osmium tetraoxide (Sigma-Aldrich 201030) in the same buffer for 1 h and washed with PBS before gradient dehydration in 50, 70, 90, and 100% ethanol, keeping each for 15 min, at room temperature (RT). Then tertiary butanol was used to collect samples, which were placed on round 0.5-cm transparent glass slides, for permanent fixation before drying and mounting on metallic stubs. The preparation was coated with gold. The acceleration voltage was fixed at 3.5 kV. High-resolution scanning electron microscope (HR-SEM) images were obtained on a Jeol JSM-7400F electron microscope at different magnifications.

## Results

### Stimulation of Fungal Propagule Release From Blastoclad and Infected Algal Cultures

The first trial for stimulation of propagule release was done according to the “wet plate” method (Ahonsi et al., 2007), previously described for zoospore mass production from the pathogenic Phytophthora and Pythium species. This method is based on *in vitro* agar plate culturing and culture flooding with distilled water, after fungal biomass production. We tested the effect of different factors on *P. sedebokerense* propagule production using the wet plate method, and the results are summarized in Table 1. We found that replacing distilled water with the mBG_11_ medium for plate flooding resulted in higher propagule production, with even higher production when mBG_11_ without NO_3_ (no NaCl added) was used. Also, the culture at the stationary stage (8–10 d) yielded more propagules than did the culture at the logarithmic growth phase (3–6 d). Additionally, illuminating the plates for a short time (4 h), with high light intensity (160 μmol photon m^−2^ s^−1^), prior to the incubation in dim light, yielded much higher propagule production. Incubating the illuminated culture afterwards at either 25° or 30°C in dim light resulted in the same propagule yields.

**Table 1:** Effect of biotic and abiotic factors applied separately on *P. sedebokerense* propagule production based on the “wet plate” method modification.

**Tested factor**		**Efficacy of treatment**
Flooding solution	Distilled water	(+)
	mBG_11_	(++)
	mBG_11_ without NO_3_ and no NaCl added	(+++)
Culture age	3–6 days	(+)
	8–10 days	(++)
Illumination^*^	Dim light	(+)
	4 h high light before dim light	(+++)
Temperature (°C)	25	(+)
	30	(+)
Stratification at 4°C	Not applied	(+)
	30 min	(+++)

*“+” symbol in brackets indicates efficacy of treatment*.

* *150–170 μmol photon m^−2^ s^−1^*.

Stratification in seed science/biology is the process of pretreating seeds to simulate the natural conditions that a seed must endure before germination. We have applied this method, i.e., incubating the culture for a short time on ice and subsequently at 25° or 30°C, and found it to be an efficient treatment, resulting in a larger amount of propagules, as compared to the incubation at 25° or 30°C alone (Table 1). Similar results were obtained when we applied the treatments to *P. sedebokerense* cultures grown in the liquid BGM medium. Since we could easily handle large biomass quantities in liquid cultures, and repeat the treatments several times, we routinely used liquid cultures for the induction and isolation of *P. sedebokerense* propagules. It is worth mentioning that the stationary liquid culture taken from the shaker and kept for 1 month on a bench at room temperature reduced its ability to produce propagules.

Finally, all the different treatments that separately led to a high level of fungal propagule production were combined and resulted in propagule release from all sporangia in six pulses, with an interval of 12 h between sequential propagule stimulation and harvest periods (filtration and centrifugation). Our optimal method for propagule isolation from the blastoclad cultures is, briefly, as follows: the culture at the stationary stage was transferred to an mBG_11_ medium without sodium nitrate and no addition of sodium chloride, designated the PSM and kept on ice for 25–30 min. Then flasks were illuminated with white light (150–170 μmol photon m^−2^ s^−1^) for 4 h in the shaker (150 rpm) at 25°C. Subsequently, the culture was returned to the original blastoclad growth conditions, i.e., dim light and 30°C. This method was also efficient for propagule isolation from the infected algal culture, with the exception that in this case, sporangia were taken from a 4-d-old infected culture.

**Figure 1:**
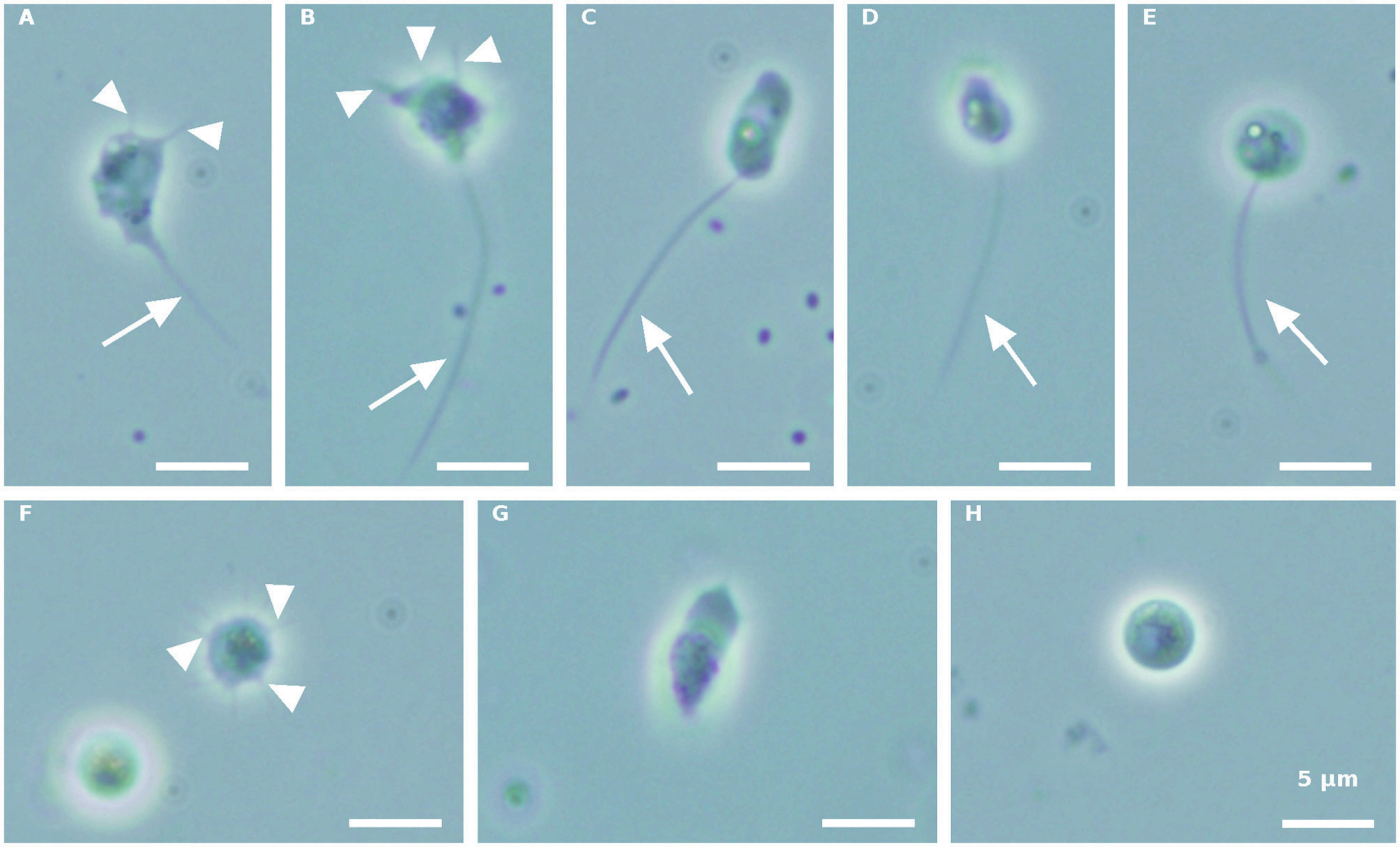
*Paraphysoderma sedebokerense (P. sedebokerense)* propagule forms obtained from both blastoclad and *Haematococcus pluvialis (H. pluvialis)* co-cultures, in the first collection. **(A,B)** Intermediate amoeboid swarmer with two **(A)** and three **(B)** visible pseudopodia and one long pseudocilium. **(C)** Elongated posteriorly flagellated zoospore. **(D)** Motile fast moving zoospore. **(E)** Round-shaped zoospore. **(F)** Multipseudopodia. **(G)** Frequently occurring amoeboid swarmer. **(H)** Spherical coatless spore. Arrowheads: pseudopodia. Arrow: pseudopodia. Scale bar: 5 μm.

Morphologically distinct propagule forms characterized the first propagule release (Figure 1). Intermediate amoeboid-form cell bodies, with two (Figure 1A) or three (Figure 1B) visible pseudopodia (arrowhead) and one long pseudocilium (arrow), fast running posteriorly flagellated zoospores with different shapes (Figures 1C–E), and multipseudopodia (Figure 1F), were observed under the microscope, in both the blastoclad and infected algal cultures. Nevertheless, amoeboid swarmers (Figure 1G) and round 3.7–5-μm-sized coatless spores (Figure 1H) dominated at the beginning of propagule release (first harvest, see also **Figure 3A**). However, when the infected algal culture was heavily contaminated with microbes, the amoeboid forms were bigger than the regular amoeboid swarmers obtained from the axenic blastoclad culture (data not shown).

**Figure 2:**
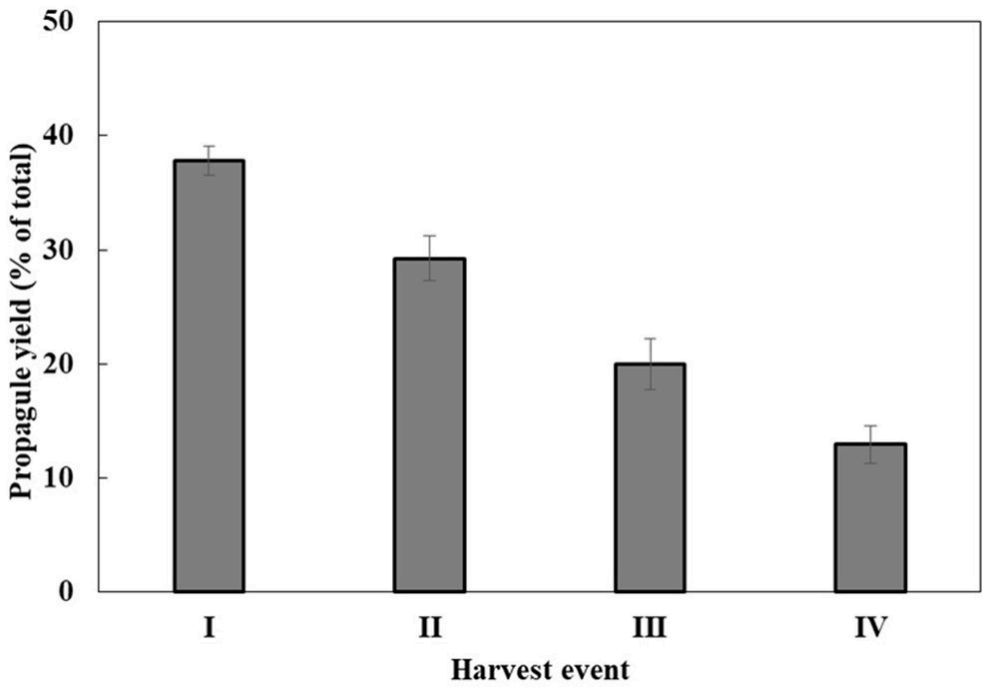
*P. sedebokerense* propagule yields during four sequential harvests. The wet weight biomass obtained in each harvest out of the total wet weight biomass obtained in all four harvests (100%) was calculated. Values represent the average of three independent experiments ± SE.

**Figure 3:**
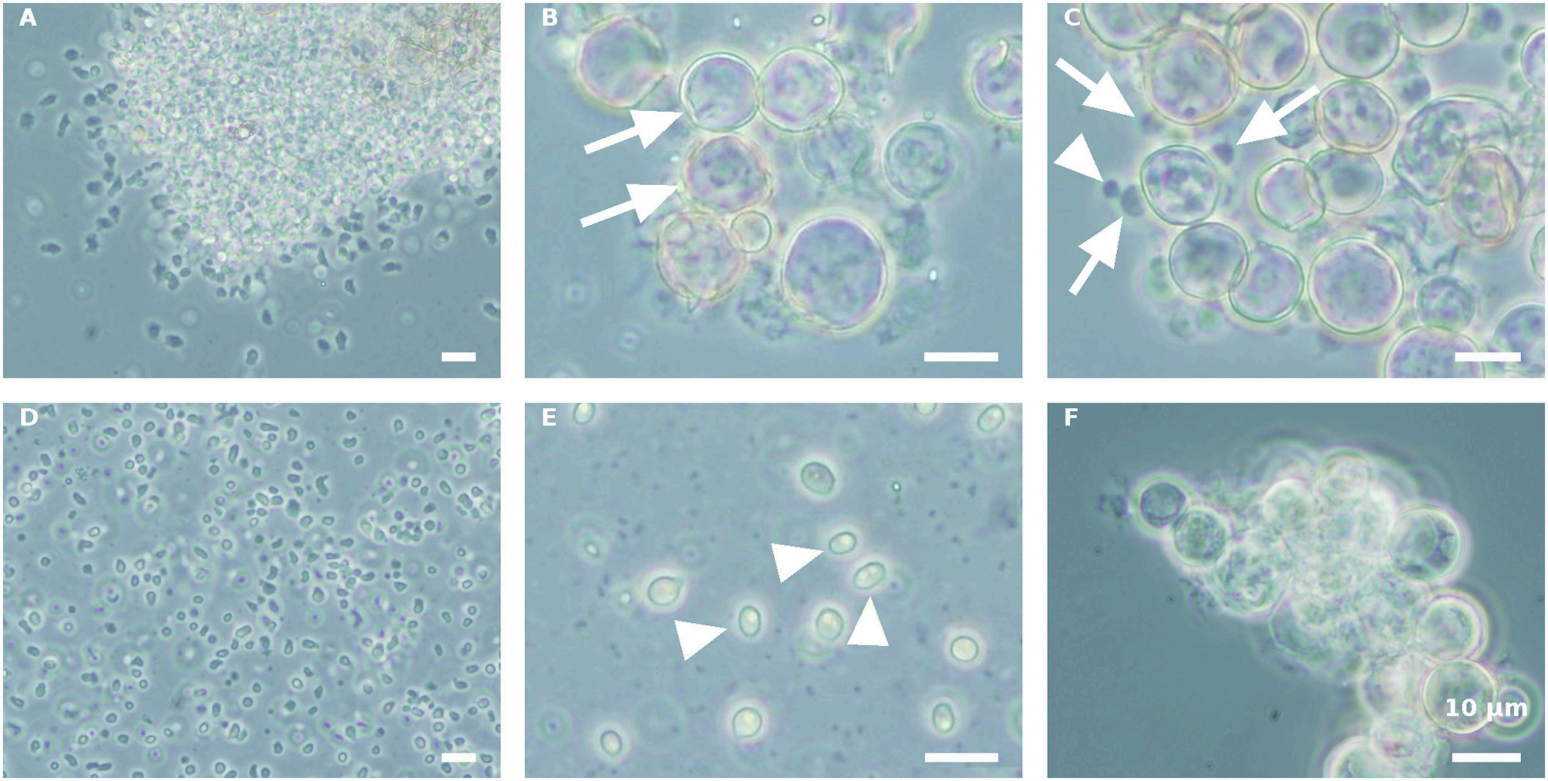
Stimulation of *P. sedebokerense* propagule release and their fate in PSM and BGM media. **(A)** The culture after the first stimulation of propagule release and before harvesting is dominated by amoeboid swarmers. **(B)** Empty (arrows) sporangia after propagule release in PSM after the fourth harvest. **(C)** Secondary propagule encystment (arrowhead) on primary cyst (arrow) in PSM. **(D)** Isolated propagules right after harvest. **(E)** Collapsed propagules in ellipsoid forms (arrowhed), after inoculation in PSM for 5 d. **(F)** Normal sporangium development of isolated propagules after inoculation in BGM for 5 d. Scale bar: 10 μm.

Propagule harvest was done 3–4 times, with an ~12-h interval between harvests. The yield of propagules in each harvest linearly decreased during subsequent harvesting to about 13% of the total propagule yield (calculated on the basis of wet biomass) in the fourth harvest (Figure 2). At the end of the sporogenesis, which corresponds to the sixth propagule stimulation, almost all sporangia were empty (Figure 3B). The final fifth and sixth propagule stimulations were characterized by small-sized (~3 μm) round spores, with a small amount of propagule biomass; thus, they were not harvested. All types of propagules, including small round wall-less spores, were able to infect the algal cultures. It is worth mentioning that if the propagules were not separated from the sporangial biomass on their day of release, they quickly attached to the sporangia's cell walls, no matter whether it was an empty or a full sporangium (Figure 3C). Further development of such propagules (encystment or collapse) on the sporangial cell wall depends on the environmental conditions. Secondary spore (Figure 3C, arrowhead) attachment and further encystment on a primary spore (Figure 3C, arrow) were frequently observed in PSM, when the harvest of propagules was delayed. In contrast, harvesting the propagules (Figure 3D) and re-inoculating them in the same medium (PSM) resulted in their transformation into bright, non-infectious ellipsoid spores (Figure 3E). However, inoculating the propagules in the BGM medium, under the same conditions (30°C, dim light, 150 rpm), led to normal sporangial development (Figure 3F).

### Inoculation of Isolated *P. sedebokerense* Propagules With an *H. pluvialis* Culture Medium

**Figure 4:**
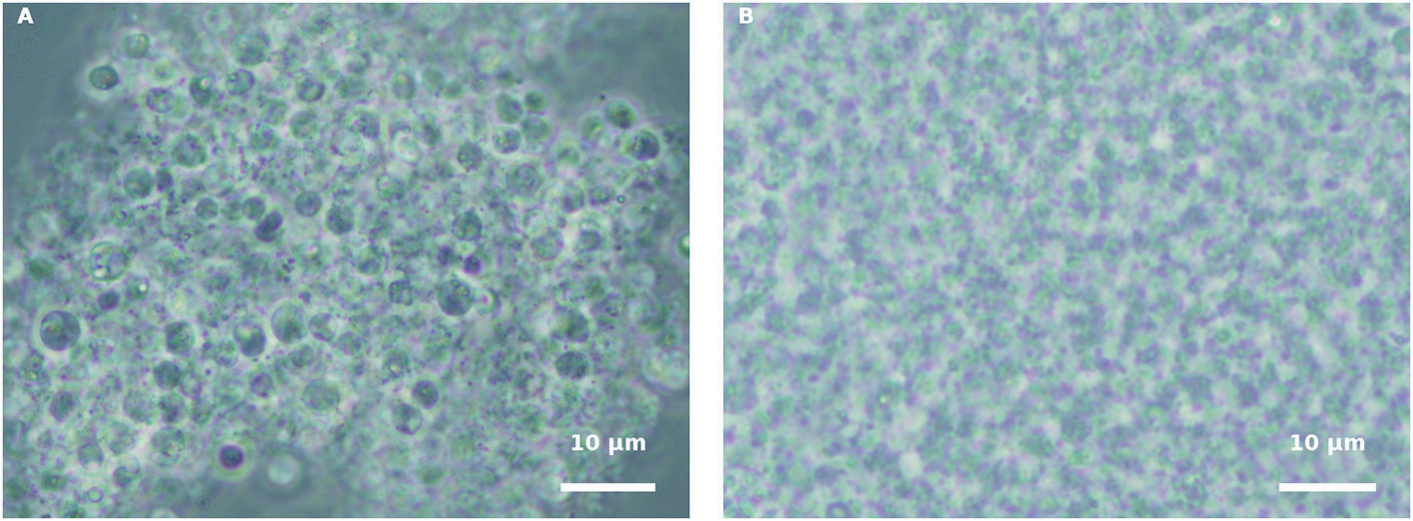
Incubation of isolated propagules derived from blastoclad culture with algal supernatant, collected from 7-d-old green *H. pluvialis* culture. **(A)** Spores of blastoclad 11 HAI. **(B)** Decomposition of propagules at 30 HAI. Scale bar: 10 μm.

In order to understand whether the green *H. pluvialis* culture supernatant at 7 d of growth alone had any influence on propagule encystment, harvested propagules, originating from either a pure fungal culture or an infected *H. pluvialis* culture, were inoculated with an algal supernatant, and conditions were kept similar to those of fungi cultivation. Similar results were observed in both propagule preparations. Eleven hours after inoculation (HAI), propagules changed their shape into round spores, and aggregates of developed round spores were evident (Figure 4A). However, cyst cell wall formation, which usually occurs during host-parasite interaction, was not observed in the propagule culture “nourished” by the algal supernatant. In contrast, these round spores disintegrated later on, as was evident by microscopic observation 30 HAI (Figure 4B). A similar phenomenon was observed when propagules were inoculated in a fresh mBG_11_ NR medium (data not shown). The inoculation of such suspensions with the intact algal culture did not cause any infection.

### Inoculation of Isolated *P. sedebokerense* Propagules With *H. pluvialis* Cultures

Isolated propagules derived either from a *P. sedebokerense* culture alone or after co-culturing with *H. pluvialis* were harvested and used for a set of experiments, to test the effect of specific environmental factors on host-parasite interaction. We noticed that, in a concentrated preparation/suspension of the fungal propagules, aggregation occurred very quickly. To avoid this situation and to allow us to study recognition, host attachment and further infection processes, a calibration of the ratio between host and parasite was first established. A ratio of 1 mg (wet weight) of the *H. pluvialis* green culture (1.1–1.5 × 10^5^ cells) and 0.25 mg (wet weight) collected *P. sedebokerense* propagules (6.74 × 10^6^ cells), in a final volume of 15 mL, was found to be optimal to allow infection but not aggregation of propagules.

Both intact and autoclaved inactivated red and green algal cells were inoculated with *P. sedebokerense* propagules that were obtained from both synthetic (pure blastoclad culture) and infected algal cultures, in both the presence and absence of nitrate. The results described hereafter were similar using both types of isolated propagules, no matter from which culture they were obtained. The rationale behind testing autoclaved cells stems from our previous finding proposing a sugar moiety of Lac-Nac on the *H. pluvialis* cell wall as the target of recognition by the parasite *P. sedebokerense* (Gutman et al., 2011). We assumed that such a molecule is heat-resistant, and therefore, we used autoclaved cells in this study. Indeed, in preliminary infection tests (not shown), we detected cysts on autoclaved cells.

**Figure 5:**
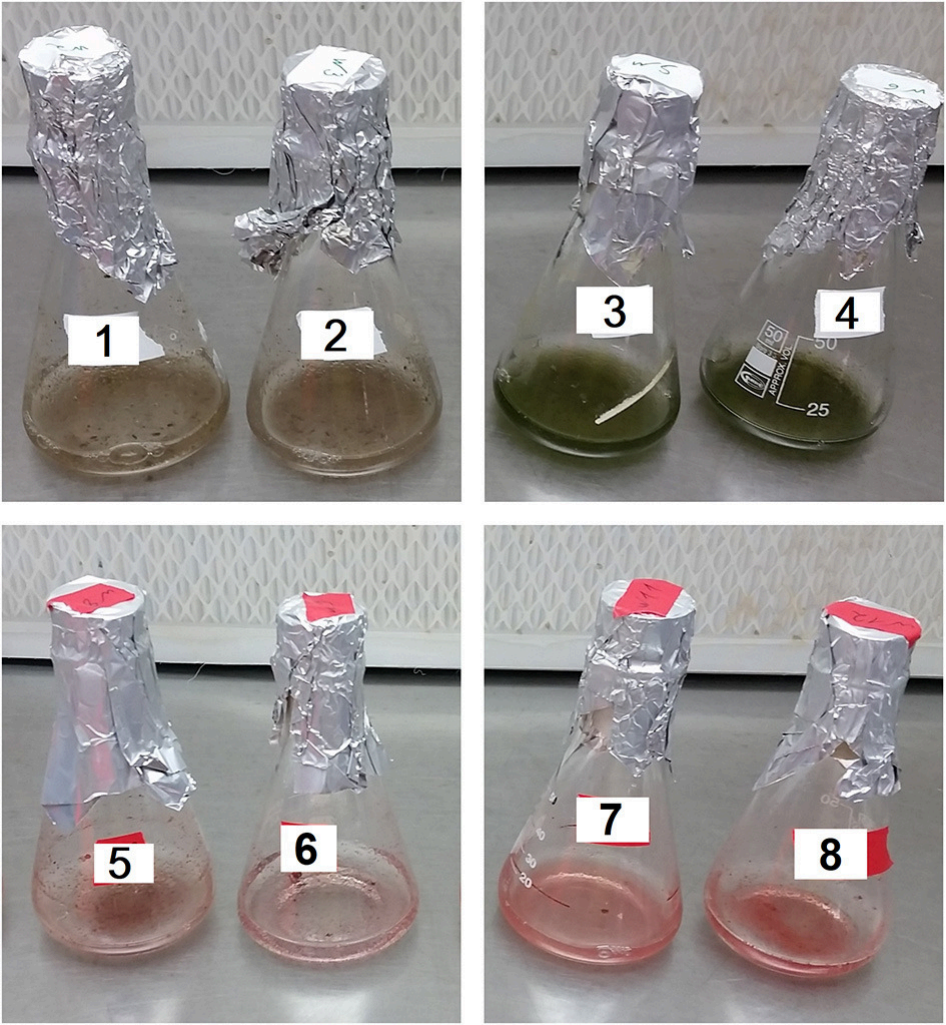
Live **(1,2)** and autoclaved **(3,4)** green *H. pluvialis* vs. live **(5,6)** and autoclaved **(7,8)** red cultures inoculated with isolated *P. sedebokerense* propagules under different conditions. **(1,3,5,7)**: Fresh nitrogen-replete mBG_11_ medium. **(2,4,6,8)**: Fresh nitrogen-depleted mBG_11_ medium. Pictures were taken 132 HAI.

**Figure 6:**
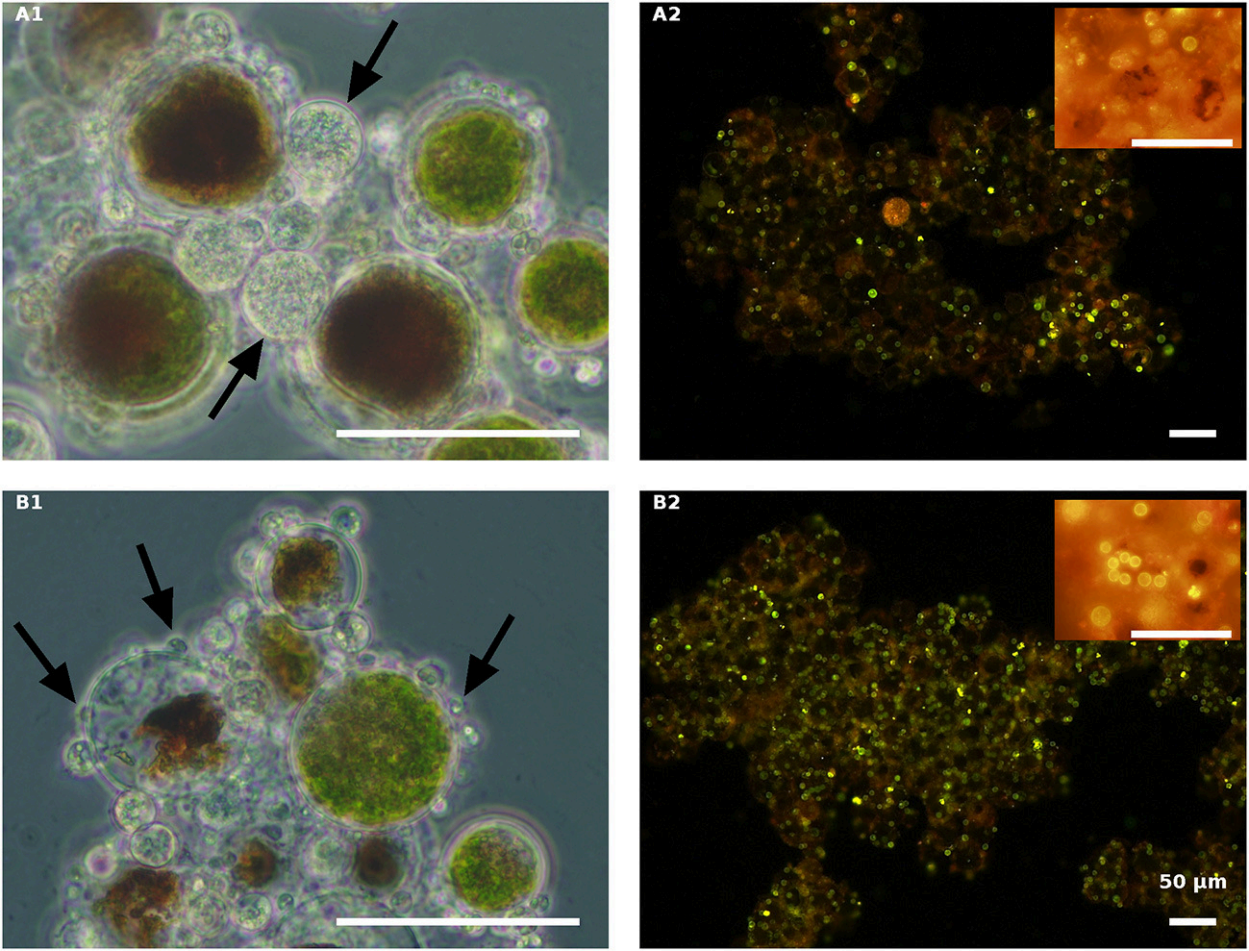
Live green *H. pluvialis* cultures inoculated with *P. sedebokerense* propagules in fresh nitrogen-replete **(A)** and fresh nitrogen-depleted **(B)** mBG_11_ media. Microscopic observations were carried out 84 HAI (phase contrast: left column) and 132 HAI after Nile red staining (fluorescence: right column). The pH of cultures was monitored right before inoculation and found to be: 7.0 and 6.7 for **A** and **B**, respectively. Scale bar: 50 μm. Arrows indicate big and small cysts in **A1** and **B1**, respectively.

**Figure 7:**
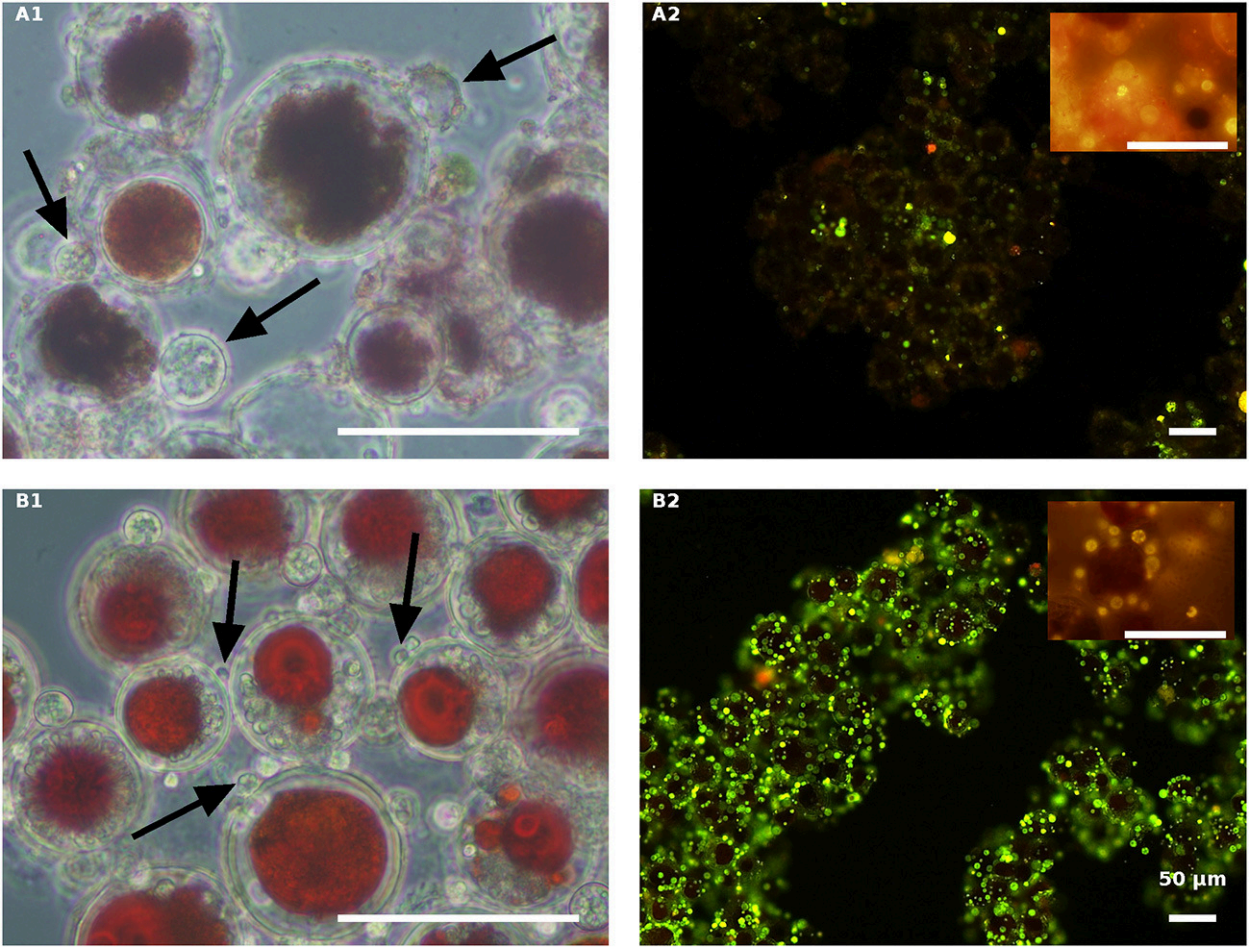
Live red *H. pluvialis* cultures inoculated with *P. sedebokerense* propagules in fresh nitrogen-replete **(A)** and fresh nitrogen-depleted **(B)** mBG_11_ media. Microscopic observations were carried out 84 HAI (phase contrast: left column) and 132 HAI (fluorescence Nile red staining: right column). The pH of cultures was monitored right before inoculation and found to be: 7.1 and 6.7 for **A** and **B**, respectively. Scale bar: 50 μm. Arrows indicate big and small cysts in **A1** and **B1**, respectively.

**Figure 8:**
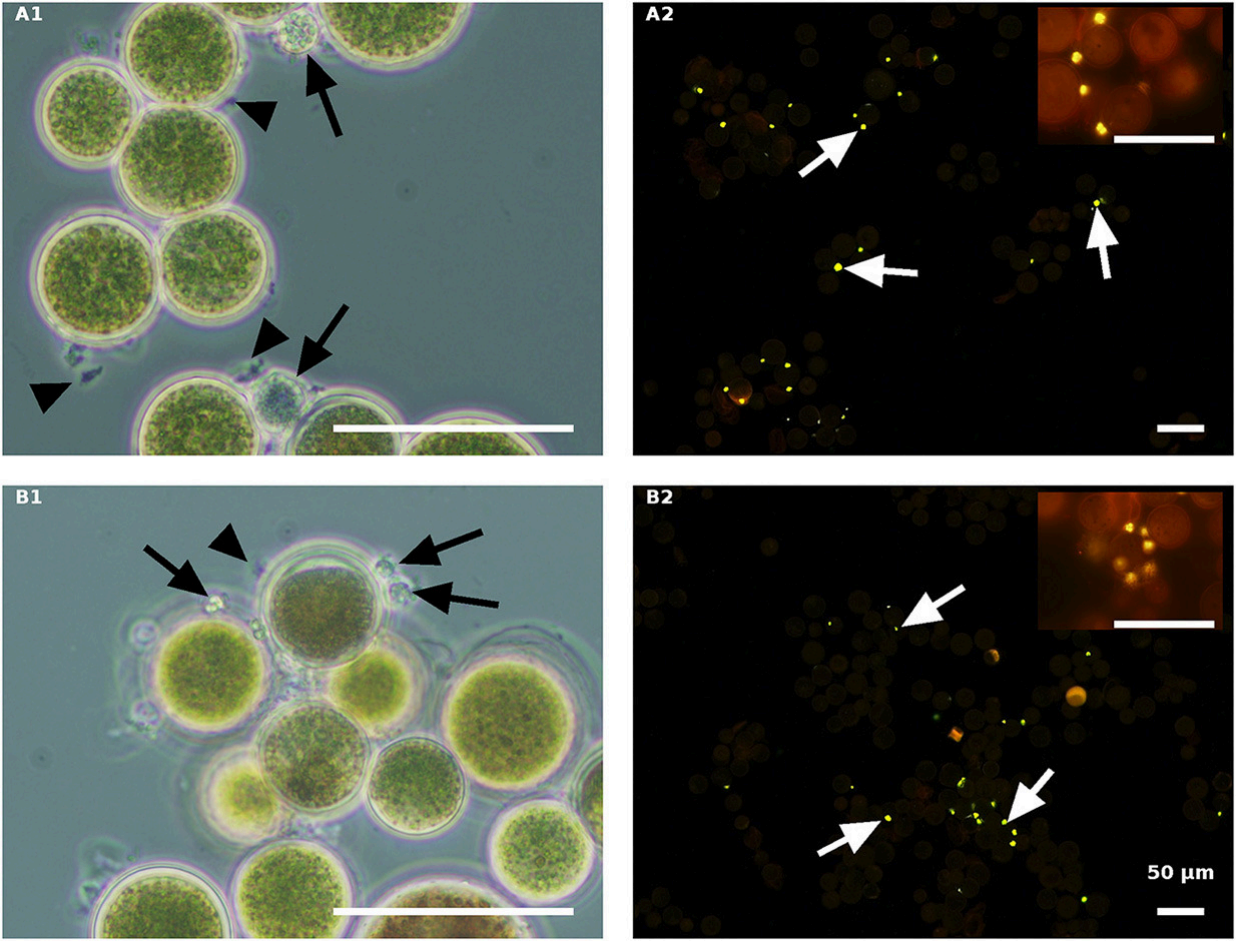
Autoclaved green *H. pluvialis* cultures inoculated with *P. sedebokerense* propagules in fresh nitrogen-replete **(A)** and fresh nitrogen-depleted **(B)** mBG_11_ media. Microscopic observations were carried out 84 HAI (phase contrast: left column) and 132 HAI (fluorescence Nile red staining: right column). The pH of cultures was monitored right before inoculation and found to be: 6.7 and 7.0 for **A** and **B**, respectively. Scale bar: 50 μm. Black arrowheads indicate amoeboid swarmers; black arrows indicate cysts; white arrows indicate oil droplets in small undeveloped cysts.

**Figure 9:**
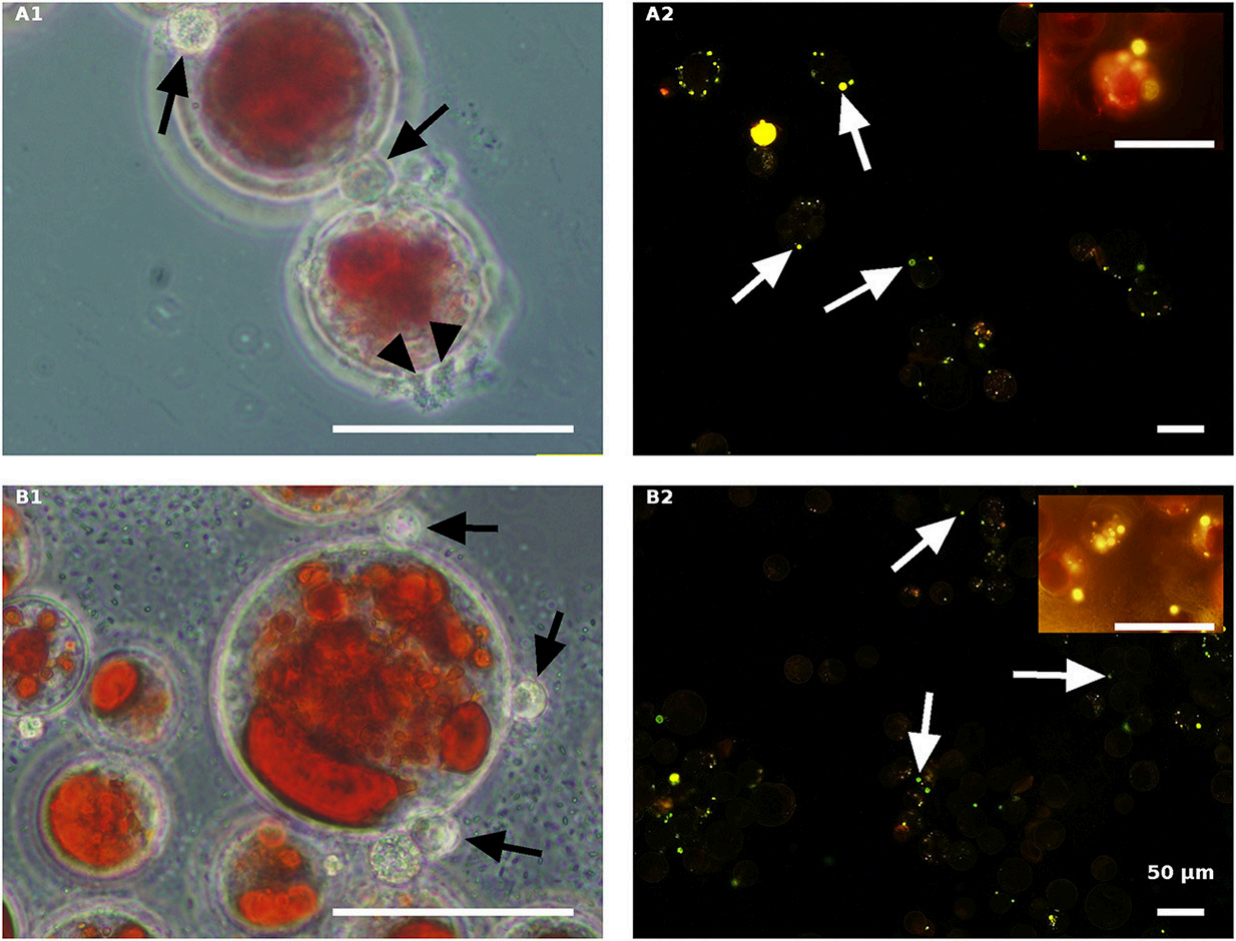
Autoclaved red *H. pluvialis* cultures inoculated with *P. sedebokerense* propagules in fresh nitrogen-replete **(A)** and fresh nitrogen-depleted **(B)** mBG_11_ media. Microscopic observations were carried out 84 HAI (phase contrast: left column) and 132 HAI (fluorescence Nile red staining: right column). The pH of cultures was monitored right before inoculation and found to be: 6.9 and 6.7 for **A** and **B**, respectively. Scale bar: 50 μm. Black arrowheads indicate amoeboid swarmers; black arrows indicate cysts; white arrows indicate oil droplets in small undeveloped cysts.

After inoculating the host with *P. sedebokerense* propagules, at the above mentioned ratio (a biomass ratio of 1:4, w/w), the infection process at 30°C was followed by macro- (Figure 5) and microscopic (Figures 6–9) observations.

In the live green *H. pluvialis* cultures, inoculated with propagules in either a fresh mBG_11_ NR (Figure 5, no. **1**) or a fresh mBG_11_ ND medium (Figure 5, no. **2**), biomass clump formation and a color change from green to brown were observed in cultures 132 HAI, indicating that progressive infection developed. In contrast, the autoclaved green cells under the same conditions (Figures 5, nos. **3**,**4**) did not change their culture color or homogeneity.

Similar to the live green cultures, the live red *H. pluvialis* cultures, inoculated with propagules in either the NR (Figure 5, no. **5**) or the ND (Figure 5, no. **6**) fresh mBG_11_ medium, changed their color from vivid red to pallid red, turned clumpy, and settled quickly at the bottom of the flask, 132 HAI. These changes indicate the development of a devastating infection. In contrast, the autoclaved red cultures, inoculated with propagules under the same conditions, behaved similarly to the autoclaved green cultures, and did not change their homogeneity or color in either condition (Figures 5, nos. **7**,**8**), as observed by the naked eye.

Samples from all the above cultures were taken for light microscopic observations 84 HAI (Figures 6–**9A1,B1**) and for Nile red oil globules staining and infection determination under a fluorescence microscope 132 HAI (Figures 6–**9A2,B2**).

As indicated above, live cultures of *H. pluvialis*, independent of the N presence in the medium (ND or NR) and the physiological state of the cells (green or red), inoculated with *P. sedebokerense* propagules, developed epidemics that finally led to the collapse of the culture (Figure 5). Microscopic observations of green cells 84 HAI showed no significant differences between the ND and NR cultures (Figures 6A1,B1). However, at a later stage (132 HAI), staining fungal oil bodies with Nile red as an indicator for fungal growth revealed differences between treatments. In the ND medium, fewer cysts were observed under the microscope (Figure 6A2), while in the absence of nitrogen, the cysts were more abundant and contained more oil bodies, as indicated by the more intense Nile red yellow fluorescence (Figure 6B2).

The effect of nitrogen was very clear in the live red algal culture cultivated with the propagules (Figure 7). In the live red cultures, where nitrogen was sufficient, infection developed more quickly, as was evident by the change from the vivid red color of the algal cells to red-brown 84 HAI in the NR variant but not in the ND variant (Figures 7A1,B1). Moreover, observations at a later stage (132 HAI) revealed faint Nile red staining in the NR but not in the ND infected culture, indicating the occurrence of fewer oil bodies (fewer resting cysts) in the NR culture (Figure 7A2) than in the ND variant (Figure 7B2).

In contrast to the live cells, the autoclaved green cells, both in the NR (Figure 8A) and the ND (Figure 8B) media 84 HAI, still had active inoculated amoeboid propagules (arrowhead) and only a certain number of medium-sized *P. sedebokerense* cysts (arrow) attached to the host. Observations at a later stage (132 HAI) with Nile red staining revealed only very few cysts with dispersed lipid globules, without any evidence for sporangium development or propagule release in either the NR or ND culture, indicating the collapse of cysts on the autoclaved green cells.

The autoclaved red algal variants (Figure 9) were all similar with rare cyst development 84 HAI (Figures 9A1,B1) and no further development (collapse) at a later stage (132 HAI) (Figures 9A2,B2). These results are quite similar to those obtained with the autoclaved green cells described above, indicating the ability of propagules to encyst on the autoclaved cells without further development.

### Scanning Electron Microscopy (SEM)

Fungal-infected Haematococcus cells were harvested for SEM analysis, to gain insight into the host-parasite recognition process and to clarify the type of interaction between the two. In an effort to study and distinguish the attachment event occurring in the host and parasite interaction, live green and red cultures, as compared to their autoclaved variants, were observed by SEM at early and late stages of infection.

**Figure 10:**
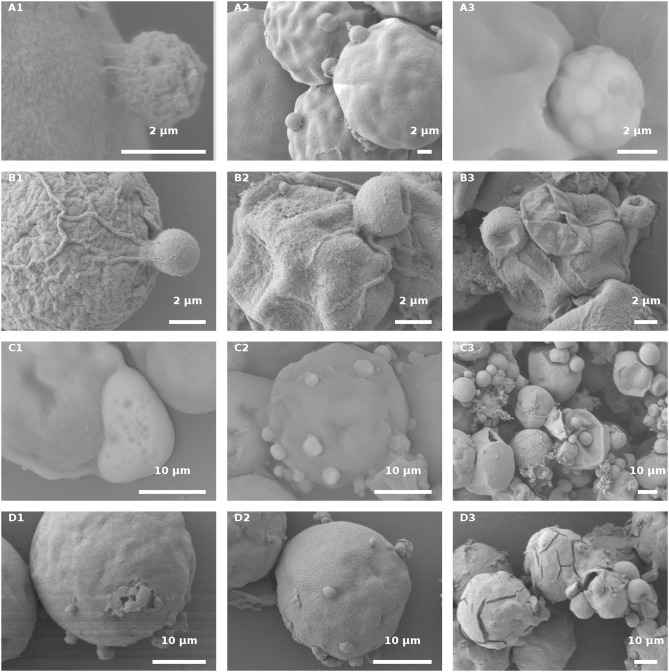
*H. pluvialis* and *P. sedebokerense* propagule interaction studies using SEM. **(A)** Infected live green *H. pluvialis* culture; a cyst on the host with a visible sticky area between the two, at an early stage of the encystment process (30 HAI) in higher **(A1)** and lower **(A2)** magnifications. **(A3)** *P. sedebokerense* sporangium with visible spores inside of it, 24 HAI. **(B)** Propagule encysted on autoclaved green *H. pluvialis* cells **(B1)**, which later collapsed (30 HAI, **B2,B3**). **(C)** Infected live red *H. pluvialis* culture; **(C1)** amoeboid propagule crawling on the host, 24 HAI; **(C2)** algal cell carrying several encysted propagules, 36 HAI; **(C3)** *H. pluvialis* culture heavily infected by *P. sedebokerense*, 96 HAI. **(D)** Propagule encysted on autoclaved red *H. pluvialis* cells, which later collapsed; two amoeboid swarmers at an early host recognition stage **(D1)** encysted propagules **(D2)**, and collapsed cysts 30 HAI **(D3)** under high resolution; cracks in the cell wall of *H. pluvialis* can be observed.

In the infected culture of live green *H. pluvialis*, a sticky area was observed at the attachment point between the host and parasite, at early stages of encystment (Figure 10A1). We assume that the production of such sticky substances is mandatory and represents the initial stage in the recognition process as those sticky substances were seen between all host-parasite “mating” events (Figure 10A2). The cysts' further development into fully round sporangia on the host cell wall, with visible spore development inside the sporangia, were observed in live green cells starting from 24 HAI (Figure 10A3).

Similar sticky zones of parasite propagule attachment to the host were also observed in the infected non-living green algal culture. However, the propagule surfaces were smoother (Figure 10B1) than those of the propagules attached to the live green algal cells. Later on (30 HAI), SEM observations revealed very tiny undeveloped cysts, which lost their roundness and changed into one-sided shrunken flattened cells (Figures 10B2,B3).

In infected live red algal cultures, at the beginning stage of the infection (24 HAI), amoeboid propagules were visible (Figures 10C1); similar amoeboid propagules were also visible in the live green algal culture (not shown). As compared to the infected live green algal cells (Figure 10A2), most of the cells carried more cysts per host cell 30 HAI (Figure 10C2). At a later infection stage (96 HAI), cysts were smaller and more abundant as compared to the ones carried by live green algal cells, and no sporangium development was observed (Figure 10C3).

The autoclaved red cells at the beginning of infection showed proper host-parasite “mating” events, i.e., ameoboid attachment to host cell walls (Figure 10D1) and small-sized cyst development (Figure 10D2), 30 HAI. There were many host cells that had visible cracks in their cell walls, which were absent in the autoclaved green cells (Figure 10D3). The cracks were further confirmed by full extraction of the pigments with acetone from the autoclaved red cells but not from the green intact or autoclaved cells (Figure S1). The cracked red cells do not support penetration of fungal zoospores through the cracks into the host cell cytoplasm, or their feeding and development within the cell (Figure 10D3). In this culture, only a few cyst-carrying autoclaved red cells were observed, and the cysts shrank as the time of infection progressed, 30 HAI (Figure 10D3).

## Discussion

The interaction between the phyco-parasite *P. sedebokerense* and its host *H. pluvialis* starts when the host is recognized by the parasite's propagules. Therefore, a method to isolate a large amount of propagules is of great importance for the investigation into host-parasite interactions. For the first time, we report here an efficient method for stimulating propagule release from both a pure *P. sedebokerense* fungal culture and from its infected algal host culture, and we further describe a method to isolate them.

It is generally accepted that nutrient limitation, such as nitrogen or carbon, stimulates sporulation (Judelson and Blanco, 2005; Pistininzi et al., 2014), by activating sporulation-induced genes. So far, nothing is known about any sporogenesis-inducing factors for *P. sedebokerense*. Abiotic factors governing the stimulation of sporogenesis in other fungal species (Soll and Sonneborn, 1971; Ribeiro, 1978; Cerenius and Söderhäll, 1985; Estrada-Garcia et al., 1989; Serrano et al., 2013; Buller, 2014; Pistininzi et al., 2014), have been examined here, for the first time, in the *P. sedebokerense* species. We have found that light, temperature, the flooding medium, and culture age are all important factors influencing the spore emergence from sporangia in *P. sedebokerense*. However, the integration of various temporal treatments, i.e., the “stratification” of a dense culture of young *P. sedebokerense* sporangia by low temperature in PSM, followed by high light exposure and then transfer to favorable conditions (dim light, 30°C), was required to gain a large amount of fungal propagules. These propagules were separated from the cysts by filtration via a 5-μm filter and were further concentrated by centrifugation.

The mechanism by which low temperature incubation positively affects sporogenesis in *P. sedebokerense* is not clear. In *Phytophthora infestans*, multiple signal transduction pathways regulate cellular events during zoosporogenesis. It was found that immersion of sporangia in chilled water either releases a germination inhibitor or hydrates a receptor on sporangial surfaces (Tani et al., 2004). Based on cDNA arrays coupled with inhibitor treatments, the following simple model was suggested: chilling reduces the fluidity of the plasma membrane, which either directly activates phospholipase C or modifies the interaction between phospholipase C and another membrane-bound protein. Phospholipase C would then lead to the activation of inositol triphosphate gated Ca^2+^ channels, which will thus increase Ca^2+^ concentrations in the cytoplasm. These changes prompt zoospore release, via the activation of kinases or phosphodiesterases responsible for zoosporogenesis (Kim and Judelson, 2003; Tani et al., 2004). This model, suggesting a major role for Ca^2+^ in sporulation, correlates well with our previous finding, demonstrating a Ca^2+^ requirement for *P. sedebokerense* epidemic development in *H. pluvialis* cultures (Gutman et al., 2011).

Simultaneous flooding and cooling facilitate zoospore release from mature sporangia, by turgor pressure (Judelson and Blanco, 2005). Although our applied propagule production method was different from the wet plate method (Ahonsi et al., 2007), in *P. sedebokerense*, primary flooding resulted in the emergence of more propagules (6.74 × 10^6^ zoospores) than previously reported for other species (Dill and Fuller, 1971; Eye et al., 1978; Appiah et al., 2005; Pistininzi et al., 2014). Re-flooding allowed us to harvest propagules several more times; however, the yield linearly decreased (Figure 2). Such repeated events of propagule release—such as spore dispersal *in situ*—are most probably an adaptation developed by pathogenic fungal species to cope with environmental conditions.

Blastocladiales are the only fungal order known to exhibit an alternation of haploid and diploid generations (James et al., 2014). According to Letcher et al. (2016), in *P. sedebokerense*, only amoeboid swarmers can be produced mitotically from vegetative (asexual) sporangia, while both amoeboid and uniflagellated zoospores can be produced by meiosis from resting (sexual) sporangia. We can thus conclude that the conditions that we used for sporangium development, both on the host and in the synthetic medium, were favorable for the parasite to develop vegetative sporangia, and therefore, mainly amoeboid swarmers were produced. However, when the culture continuously faced nitrogen depletion, cysts began to produce intermediate spores (propagules), most probably from the later developed resting sporangia. Intermediate zoosporic forms, described recently by Strittmatter et al. (2016) in an *H. pluvialis* co-culture, are transition forms between flagellated zoospores and amoeboid swarmers.

The environmental factors affecting the formation of different propagule morphotypes are not clear. Nevertheless, when a stressed red *H. pluvialis* culture was inoculated with *P. sedebokerense*, we determined a stage during the infection process where zoospores had an elongated form, contained round oil bodies and moved so fast that capturing microscopic images was almost impossible (not shown). The life span of such posterior flagellum, fast moving zoospores was very short, and the transformation into a spherical shape through shading the flagella was barely detected under the microscope. All of these morphologies were also described recently in an *H. pluvialis* co-culture by Strittmatter et al. (2016). They found similar fast moving zoospores, 3 μm in diameter size, in 1 to 2-week-old infected *H. pluvialis* cultures. Our results, together with those of Strittmatter et al. (2016), lead us to assert that such zoospore development is related to stress conditions, most probably low nitrogen availability.

In order to understand the host cell's and the parasite propagule's interaction, we tested the interaction of pure propagules with the host cells in the presence or absence of nitrogen.

In all tested combinations, we saw the vulnerability of live algal cells, no matter whether they were red stressed or green vegetative ones. In regard to environmental conditions, in red cells cultivated with the parasite in a fresh nitrogen-rich algal medium, epidemics developed more quickly than in the ND medium. Similarly, another study, conducted on the cyanobacterium *Planktothrix rubescens* (Frenken et al., 2017), showed that the chytrid *Rhizophydium megarrhizum* proliferation rate on the host (infection) increased with the availability of nitrogen. We suggest that this pattern is related to the fact that nitrogen limitation limits parasite growth (low infection rate) and its reproductive success (developing resting sporangia), although it may also alter host defense mechanisms.

Unlike the live cells, the autoclaved algal cells seemed to serve only as a specific matrix for propagule attachment and encystment, independent of the cell type or the nitrogen presence in the medium (Figures 8, 9). In contrast to the live Haematococcus cells, the autoclaved ones maintained their constituents and pigments, indicating that further development of infection in autoclaved dead host cells does not occur. After attachment and encystment, there are no further conformational and metabolic changes that can support parasite cell development on the host, since there is no signal response coming from the dead host cells. This proves that encystment is related to cell wall autoclavable-resistant molecules (carbohydrates), which is independent of nitrogen availability. These results further support our previous findings of a Lac-Nac sugar moiety on the *H. pluvialis* cell wall and its proposed role as a recognition molecule by the parasite *P. sedebokerense* (Gutman et al., 2011). However, further sporangium development and epidemics are dependent on a signal coming from the live cells; this signal is dependent on nitrogen availability.

Differences between the NR and ND media were observed only when the host was alive; in the autoclaved cells, nitrogen had no effect. Since *P. sedebokerense* cannot use nitrate as a nitrogen source (Hoffman et al., 2008), we can suggest that the host assimilates nitrogen for the parasite and only then is organic nitrogen used for the cyst development. These results are well-correlated with the collapse of the propagules in PSM and the collapse of the cysts that developed on the autoclaved cells.

SEM observations, which show the area between the host and parasite with high resolution, shed light on host-parasite recognition and attachment processes. In live cells, at the initial stages of inoculation, a sticky type linkage between propagules and the host cell wall was evident. At later stages, development of a rhizoidal system penetrating into the host cytoplasm was observed. In autoclaved cells, we determined cracks in the red cell wall, as previously reported (Mendes-Pinto et al., 2001), but not in the green cells. This reflects a difference in the mechanical strength between green and red cell walls, as reported previously (Montsant et al., 2001). In the autoclaved red cells, we did not see any events of propagule encystment in the cracks. Therefore, we can conclude that although the endogenous constituents of these host cells are physically accessible, they cannot be hydrolyzed and consumed by the parasite. In addition, we saw one-sided flat cysts, which indicate the collapse of fungal cysts during maturation. All macroscopic and microscopic observations provide evidence that the encystment is due to a specific interaction between heat-resistant algal cell ligands and parasite propagule receptors; however, further development of the cysts requires pathogen-mediated signals from a live host. These results are in accordance with our previous findings of Lac-Nac sugar moiety on *H. pluvialis* cell wall and its proposed role as a recognition molecule by the parasite *P. sedebokerense* (Gutman et al., 2009).

In conclusion, the reproducible method described herein for pure propagule culture isolation can lead to a breakthrough in the understanding of *P. sedebokerense* and *H. pluvialis* interrelationships. It can be used for downstream applications, such as transcriptome analysis, to reveal the yet uncovered molecular information related to host-parasite gene dynamics. We must note that members of the Blatocladiomycota are also responsible for a significant decline in the amphibian population in nature, and this study will benefit the research on this topic as well. As an outcome of such studies, we can expect solutions to overcome fungal infections in different systems.

## Author Contributions

AA and AZ conceived and designed the experiments, analyzed the data, and wrote and revised the publication. AA grew the microalgae, fungi, and performed the experiments. SB supervised and provided financial support. All authors approve this work for publication.

### Conflict of Interest Statement

The authors declare that the research was conducted in the absence of any commercial or financial relationships that could be construed as a potential conflict of interest.

## Supplementary Material

Figure S1 Leaching of pigments from *Haematococcus pluvialis* cells, before and after autoclaving. Both live **(A,B)** and autoclave-inactivated cells **(C,D)** were extracted twice with acetone; pigments were fully recovered only from autoclaved red cells, and the pellet became colorless. Green **(A,C)** and red **(B,D)** *H. pluvialis* cells.

## Abbreviations

ND: nitrogen-deprived
NR: nitrogen-replete
BGM: blastoclad growth medium
PSM: propagule stimulation medium
HAI: hours after infection.

## References

[B1] Ahonsi M. O. Banko T. J. Hong C. X. (2007). A simple *in-vitro* wet-plate method for mass production of *Phytophthora nicotianae* zoospores and factors influencing zoospore production. J. Microbiol. Methods 70, 557–560. 10.1016/j.mimet.2007.06.012 17683817

[B2] Appiah A. A. Van West P. Osborne M. C. Gow N. A. R. (2005). Potassium homeostasis influences the locomotion and encystment of zoospores of plant pathogenic oomycetes. Fungal Genet. Biol. 42, 213–223. 10.1016/j.fgb.2004.11.003 15707842

[B3] Boussiba S. Vonshak A. (1991). Astaxanthin accumulation in the green alga *Haematococcus pluvialis*. Plant Cell Physiol. 32, 1077–1082. 10.1093/oxfordjournals.pcp.a078171

[B4] Buller N. B. (2014). Bacteria and Fungi From Fish and Other Aquatic Animals: A Practical Identification Manual, 2nd Edn., Oxfordshire: CABI Publishing, 881.

[B5] Carney L. T. Lane T. W. (2014). Parasites in algae mass culture. Front. Microbiol. 5, 1–8. 10.3389/fmicb.2014.00278 24936200 PMC4047527

[B6] Cerenius L. Söderhäll K. (1985). Repeated zoospore emergence as a possible adaptation to parasitism in aphanomyces. Exp. Mycol. 9, 259–263. 10.1016/0147-5975(85)90022-2

[B7] Dill B. C. Fuller M. S. (1971). Amino acid hnmobilization of fungal motile cells. Arch. Mikrobiol. 78, 92–98. 10.1007/BF00409091 5099543

[B8] Estrada-Garcia M. T. Green J. R. Booth J. M. White J. G. Callow J. A. (1989). Monoclonal antibodies to cell surface components of zoospores and cysts of the fungus *Pythium-aphanidermatum* reveal species-specific antigens. Exp. Mycol. 13, 348–355. 10.1016/0147-5975(89)90030-3

[B9] Eye L. L. Sneh B. Lockwood J. L. (1978). Factors affecting zoospore production by *Phytophthora megasperma* Var. Sojae. Phytopathol. 68, 1766–1768. 10.1094/Phyto-68-1766

[B10] Frenken T. Wierenga J. Gsell A. S. van Donk E. Rohrlack T. Van de Waal D. B. (2017). Changes in N:P supply ratios affect the ecological stoichiometry of a toxic cyanobacterium and its fungal parasite. Front. Microbiol. 8:1015. 10.3389/fmicb.2017.01015 28634476 PMC5459933

[B11] Gutman J. Zarka A. Boussiba S. (2009). The host-range of *Paraphysoderma sedebokerensis*, a chytrid that infects *Haematococcus pluvialis*. Eur. J. Phycol. 44, 509–514. 10.1080/09670260903161024

[B12] Gutman J. Zarka A. Boussiba S. (2011). Evidence for the Involvement of surface carbohydrates in the recognition of *Haematococcus pluvialis* by the parasitic blastoclad *Paraphysoderma sedebokerensis*. Fungal Biol. 115, 803–811. 10.1016/j.funbio.2011.06.006 21802061

[B13] Hebert T. T. Kelman A. (1958). Factors influencing the germination of resting sporangia of *Physoderma maydis*. Phytopathology 48, 101–110.

[B14] Hoffman Y. Aflalo C. Zarka A. Gutman J. James T. Y. Boussiba S. (2008). Isolation and characterization of a novel chytrid species (Phylum Blastocladiomycota), parasitic on the green alga *Haematococcus*. Mycol. Res. 112, 70–81. 10.1016/j.mycres.2007.09.002 18222678

[B15] James T. Y. Porter T. M. Martin W. W. (2014). Blastocladiomycota, in The Mycota, Vol. 7, *Part A, Systematics and Evolution, 2nd Edn* eds David J. McLaughlin W. Spatafora J. W. (Berlin; Heidelberg: Springer-Verlag), 177–207.

[B16] James T. Y. Yoram H. Aliza Z. Sammy B. (2011). Paraphysoderma sedebokerensis. Mycotaxon 118, 177–180. 10.5248/118.177

[B17] Judelson H. S. Blanco F. A. (2005). The spores of phytophthora: weapons of the plant destroyer. Microbiol. Nat. Rev. 3, 47–58. 10.1038/nrmicro1064 15608699

[B18] Kim K. S. Judelson H. S. (2003). Sporangium-specific gene expression in the oomycete *Phytophthora infestans*. Eukaryot. Cell 2, 1376–1385. 10.1128/EC.2.6.1376-1385.2003 14665470 PMC326645

[B19] Letcher P. M. Lee P. A. Lopez S. Burnett M. McBride R. C. Powell M. J. (2016). An Ultrastructural Study of *Paraphysoderma sedebokerense* (Blastocladiomycota), an epibiotic parasite of microalgae. Fungal Biol. 120, 324–337. 10.1016/j.funbio.2015.11.003 26895861

[B20] Mendes-Pinto M. M. Raposo M. F. Bowen J. Young A. J. Morais R. (2001). Evaluation of different cell disruption process on encysted cells of *Haematococcus pluvialis*. J. Appl. Phycol. 13, 19–24. 10.1023/A:1008183429747

[B21] Montsant A. Zarka A. Boussiba S. (2001). Presence of a nonhydrolyzable biopolymer in the cell wall of vegetative cells and astaxanthin-rich cysts of *Haematococcus pluvialis* (Chlorophyceae). Mar. Biotechnol. 3, 515–521. 10.1007/s1012601-0051-0 14961323

[B22] Pistininzi M. Weiss E. Achtemeier L. Hong C. (2014). Zoospore production biology of pythiaceous plant pathogens. J. Phytopathol. 162, 69–80. 10.1111/jph.12154

[B23] Porter T. M. Martin W. James T. Y. Longcore J. Gleason E. F. H. Adler P. H.. (2011). Molecular phylogeny of the blastocladiomycota (Fungi) based on nuclear ribosomal DNA. Fungal Biol. 115, 381–392. 10.1016/j.funbio.2011.02.004 21530920

[B24] Ribeiro O. K. (1978). A Source Book of the Genus Phytophthora. Vaduz: A. R. Gantner Verlag KG, 417.

[B25] Ribeiro O. K. (1983). Physiology of asexual sporulation and spore germination in Phytophthora, in Phytophthora: Its Biology, Taxonomy, Ecology, and Pathology, eds Erwin D. C. Bartnicki-Garcia S. Tsao P. H. (St Paul, MN: American Phytopathological Society), 55–70.

[B26] Serrano M. S. Fernández-Rebollo P. De Vita P. Sánchez M. E. (2013). Calcium mineral nutrition increases the tolerance of quercus ilex to phytophthora root disease affecting oak rangeland ecosystems in spain. Agroforestry Syst. 87, 173–179. 10.1007/s10457-012-9533-5

[B27] Soll D. R. Sonneborn D. R. (1971). Zoospore germination in *Blastocladiella emersonii*. J. Cell Sci. 9, 679–699. 5148012 10.1242/jcs.9.3.679

[B28] Strittmatter M. Guerra T. Silva J. Gachon C. M. M. (2016). A New flagellated dispersion stage in *Paraphysoderma Sedebokerense*, a pathogen of *Haematococcus pluvialis*. J. Appl. Phycol. 28, 1553–1558. 10.1007/s10811-015-0700-8 27226700 PMC4851982

[B29] Tani S. Yatzkan E. Judelson H. S. (2004). Multiple pathways regulate the induction of genes during zoosporogenesis in *Phytophthora infestans*. Molecul. Plant-Microbe Interac. 17, 330–337. 10.1094/MPMI.2004.17.3.330 15000400

