# Supplementary figures and images for: Stimulation and Isolation of Paraphysoderma sedebokerense (Blastocladiomycota) Propagules and Their Infection Capacity Toward Their Host Under Different Physiological and Environmental Conditions
Source: Front Cell Infect Microbiol. 2019 Mar 27;9:72. doi: 10.3389/fcimb.2019.00072 (PMC6446968; doi:10.3389/fcimb.2019.00072)

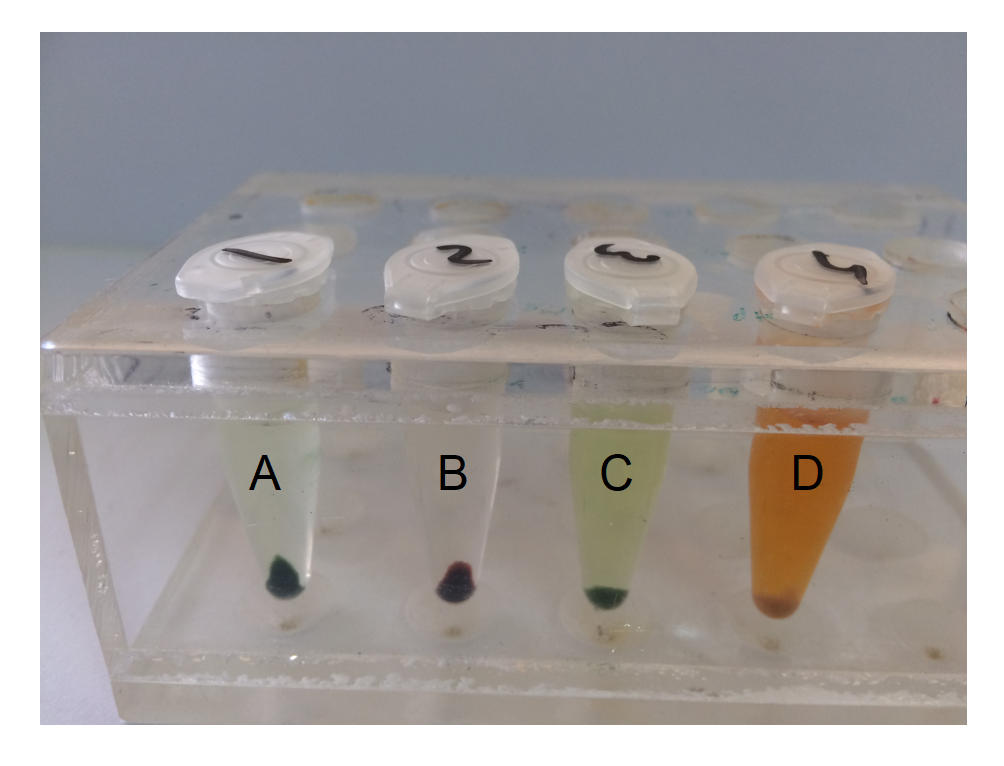

Supplement: Figure S1 — Leaching of pigments from Haematococcus pluvialis cells, before and after autoclaving. Both live (A,B) and autoclave-inactivated cells (C,D) were extracted twice with acetone; pigments were fully recovered only from autoclaved red cells, and the pellet became colorless. Green (A,C) and red (B,D) H. pluvialis cells. [file Image_1.TIF]
